# Sensitivity and specificity of tritiated thymidine incorporation and ELISPOT assays in identifying antigen specific T cell immune responses

**DOI:** 10.1186/1471-2172-8-21

**Published:** 2007-09-12

**Authors:** Vivian Goodell, Corazon dela Rosa, Meredith Slota, Beth MacLeod, Mary L Disis

**Affiliations:** 1 Center for Translational Medicine in Women's Health, 2^nd ^Floor, 815 Mercer Street, Box 358050, University of Washington, Seattle, WA, 98109 USA

## Abstract

**Background:**

Standardization of cell-based immunologic monitoring is becoming increasingly important as methods for measuring cellular immunity become more complex. We assessed the ability of two commonly used cell-based assays, tritiated thymidine incorporation (proliferation) and IFN-gamma ELISPOT, to predict T cell responses to HER-2/neu, tetanus toxoid (tt), and cytomegalovirus (CMV) antigens. These antigens were determined to be low (HER-2/neu), moderate (tt), and robustly (CMV) immunogenic proteins. Samples from 27 Stage II, III, and IV HER-2/neu positive breast cancer patients, vaccinated against the HER-2/neu protein and tt, were analyzed by tritiated thymidine incorporation and IFN-gamma ELISPOT for T cell response.

**Results:**

Linear regression analysis indicates that both stimulation index (SI) (p = 0.011) and IFN-gamma secreting precursor frequency (p < 0.001) are significant indicators of antigen specific immunity. ROC curves plotted to assess the performance of tritiated thymidine incorporation and the ELISPOT assay indicate that SI is a significant indicator of low T cell response to the HER-2/neu vaccine (p = 0.05), and of moderate and robust responses to tt (p = 0.01) and CMV (p = 0.016), respectively. IFN-gamma precursor frequency is a significant indicator of a robust T cell response to CMV (p = 0.03), but not of moderate tt (p = 0.09), or low HER-2/neu (p = 0.09) T cell responses.

**Conclusion:**

These data underscore the importance of taking into consideration the performance characteristics of assays used to measure T cell immunity. This consideration is particularly necessary when determining which method to utilize for assessing responses to immunotherapeutic manipulations in cancer patients.

## Background

A multitude of assays have been developed to measure T cell responses to a variety of antigens [1]. There is little agreement as to which method is the most superior for detecting immune responses. Antigen specific T cell proliferation as measured by tritiated thymidine incorporation and IFN-gamma ELISPOT are two of the most commonly used methods to measure T cell immunity. We questioned if there was a difference in the ability of these assays to measure a broad range of T cell responses.

In this study we utilized T cells from cancer patients that had been immunized with both a HER-2/neu protein based vaccine as well as a tetanus toxoid vaccine. Both vaccines were successful in generating antigen specific antibody immunity, an indicator of immunization. Evaluating three immunogenic proteins, HER-2/neu, tt, and CMV in these patients as representative of low, intermediate, and robust responses we determined that the proliferation assay was a better discriminator of low level immune responses than ELISPOT. These data highlight the need for immunologic monitoring core laboratories to define the performance characteristics of the methods chosen to assess the development of a T cell immune response.

## Results

### Antibody immunity to both foreign and tumor antigens is an indicator of a concomitant T cell response

**Figure 1:**
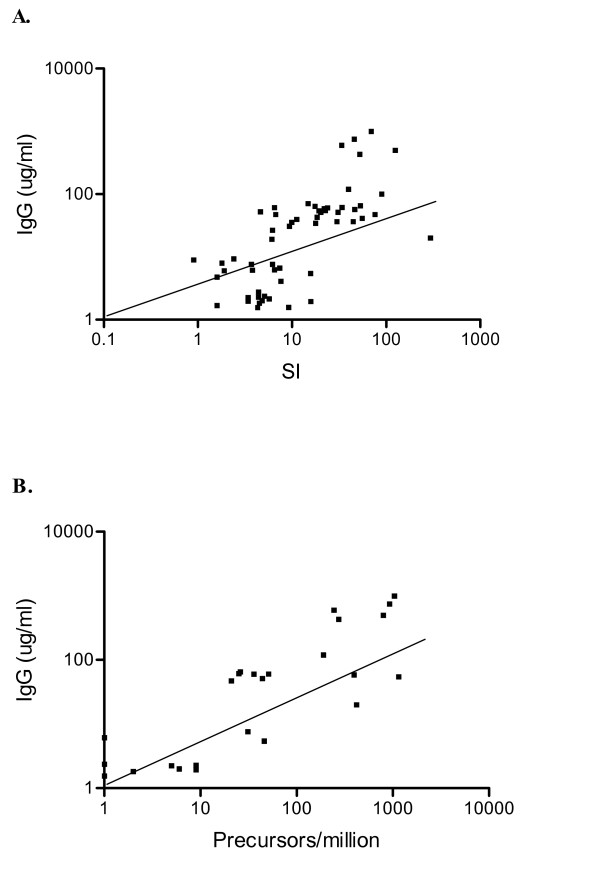
**Antibody immunity to both foreign and tumor antigens is an indicator of a concomitant T cell response**. Results from the (A) proliferation assay and (B) ELISPOT assay for antigens HER-2/neu, tt and CMV are plotted on the log-scaled horizontal axis. Antigen specific IgG antibody responses are plotted on the log-scaled vertical axis. The diagonal line indicates line of regression.

A standard measure of successful immunization is the development of antigen specific humoral immunity [2]. Furthermore, the presence of humoral immunity may serve as a marker for the presence of antigen specific CD4+ T cells [3,4]. Breast cancer patients were immunized against the HER-2/neu ICD protein and tt, and humoral immunity to these antigens measured by ELISA. Antibody responses to CMV, a viral antigen presumed to stimulate a robust immune reaction, were also measured by ELISA [5]. Successful immunization against HER-2/neu, as indicated by the presence of HER-2/neu specific antibodies, was achieved in 89% (24 of 27) of patients. Successful immunization against tt, as indicated by the presence of tt specific antibodies, was achieved in 100% of patients. Forty-four percent of the patients were CMV seropositive. Linear regression analysis of all results was performed to determine the correlation of antibody immunity to a detectable T cell response. Antibody response predicted T cell response when T cell response was assessed by either proliferation assay (Fig. 1A) or ELISPOT assay (Fig. 1B). Humoral immunity significantly (p = 0.011) predicted CD4+ T cell proliferation with an R^2 ^of 0.428, and humoral immunity significantly (p < 0.001) predicted IFN-gamma secretion with an R^2 ^of 0.531. Thus, humoral immunity and T cell immunity correlated in both foreign and tumor antigen systems.

**Figure 2:**
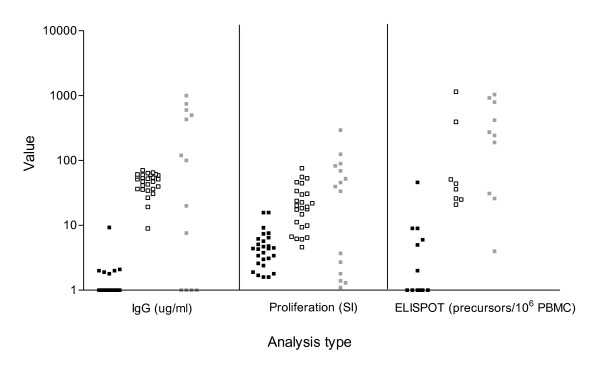
**Magnitude of T cell response differs significantly between antigens**. Boxes represent responses to the proliferation assay and ELISPOT assay for antigens HER-2/neu (closed boxes), tt (open boxes), and CMV (shaded boxes). Reporting value for the proliferation assay is SI, and reporting value for the ELISPOT assay is antigen-specific precursor/10^6 ^PBMC. Antibody immunity for the same group of subjects is shown for comparison, with a reporting value of ug/ml antigen specific IgG.

The magnitude of the T cell response differed significantly between antigens (Fig. 2). The range of T cell immunity to HER-2/neu was low to moderate with a mean SI of 5.06 (1.6–15.8) and a mean precursor frequency of 4.61 precursors/10^6 ^PBMC (0–46). Responses to tt were moderate to robust with a mean SI of 23.4 (0.9–76.2) and mean precursor frequency of 146 precursors/10^6 ^PBMC (0–1153). Responses to CMV were robust with a mean SI of 47.1 (0.7–296) and a mean precursor frequency of 329 precursors/10^6 ^PBMC (0–1037). Mean antibody responses to HER-2/neu, tt and CMV were 3.3 (0–9.3) ug/ml, 47 (8.9–70.7) ug/ml and 131 (0–1000) ug/ml, respectively. We questioned the sensitivity of these two assays, CD4+ proliferation and IFN-gamma ELISPOT, in detecting this wide range of responses.

### T cell proliferation will detect T cell immunity whether the immune response is low, moderate or robust

**Figure 3:**
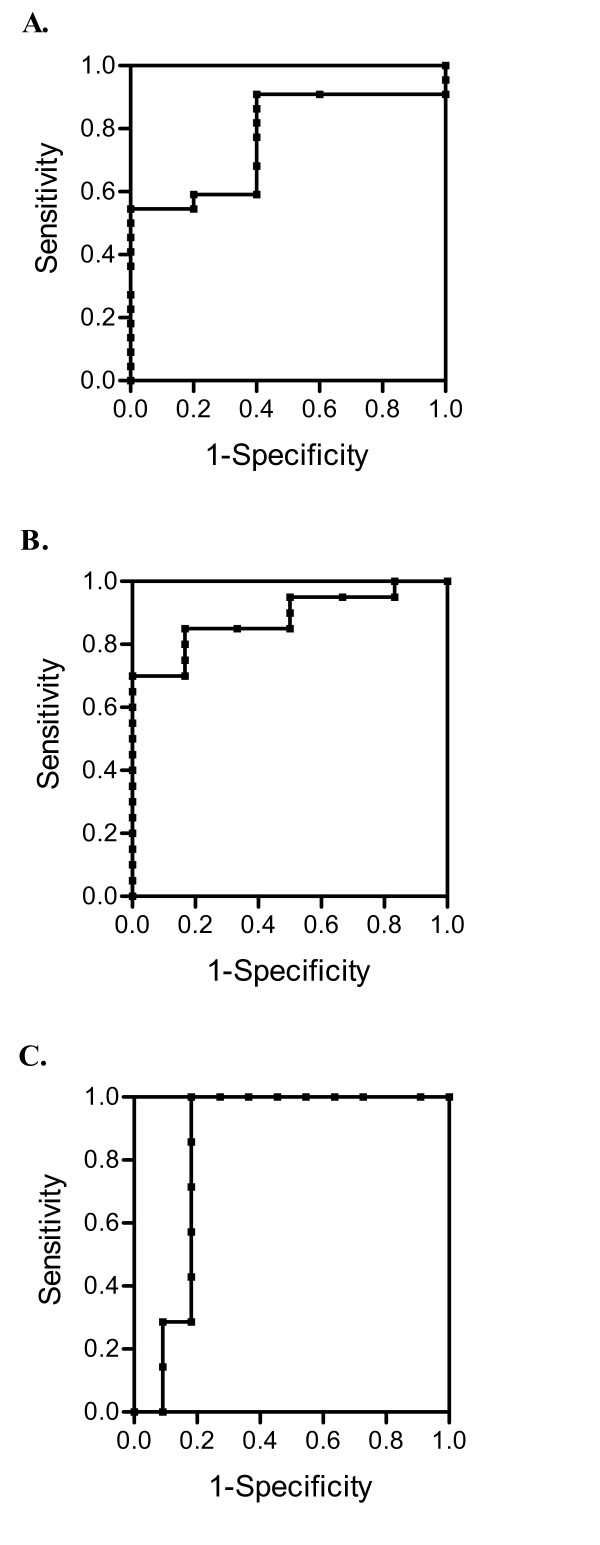
**T cell proliferation will detect T cell immunity whether the immune response is low, moderate or robust**. ROC curves were plotted to assess the diagnostic performance of the proliferation assay for (A) HER-2/neu ICD specific cellular immunity (n = 27), (B) tt specific cellular immunity (n = 18), and (C) CMV specific cellular immunity (n = 18). Samples with antigen specific circulating antibodies were considered to have positive immunity.

ROC curves were plotted to assess sensitivity and specificity of the proliferation assay, using presence of antigen specific antibodies as a marker of successful immunization. Results show that SI is a significant (p = 0.05) predictor of immunity to the HER-2/neu ICD protein with an AUC of 0.773 (Fig. 3A). This yielded a sensitivity of 83%. A subset (n = 18) of these patients was assessed by proliferation assay for cellular immunity to antigens tt and CMV. Results show that SI is a significant indicator of moderate cellular immunity to tt (AUC = 0.883, p = 0.010) (Fig. 3B) with a sensitivity of 100%, and CMV (AUC = 0.844, p = 0.016) with a sensitivity of 100% (Fig. 3C). Only 1 CMV seronegative patient had a positive CMV specific SI. Thus, the proliferation assay is capable of accurately detecting low, moderate, and robust T cell immunity.

### IFN-gamma secretion as measured by ELISPOT accurately detects robust T cell immunity

**Figure 4:**
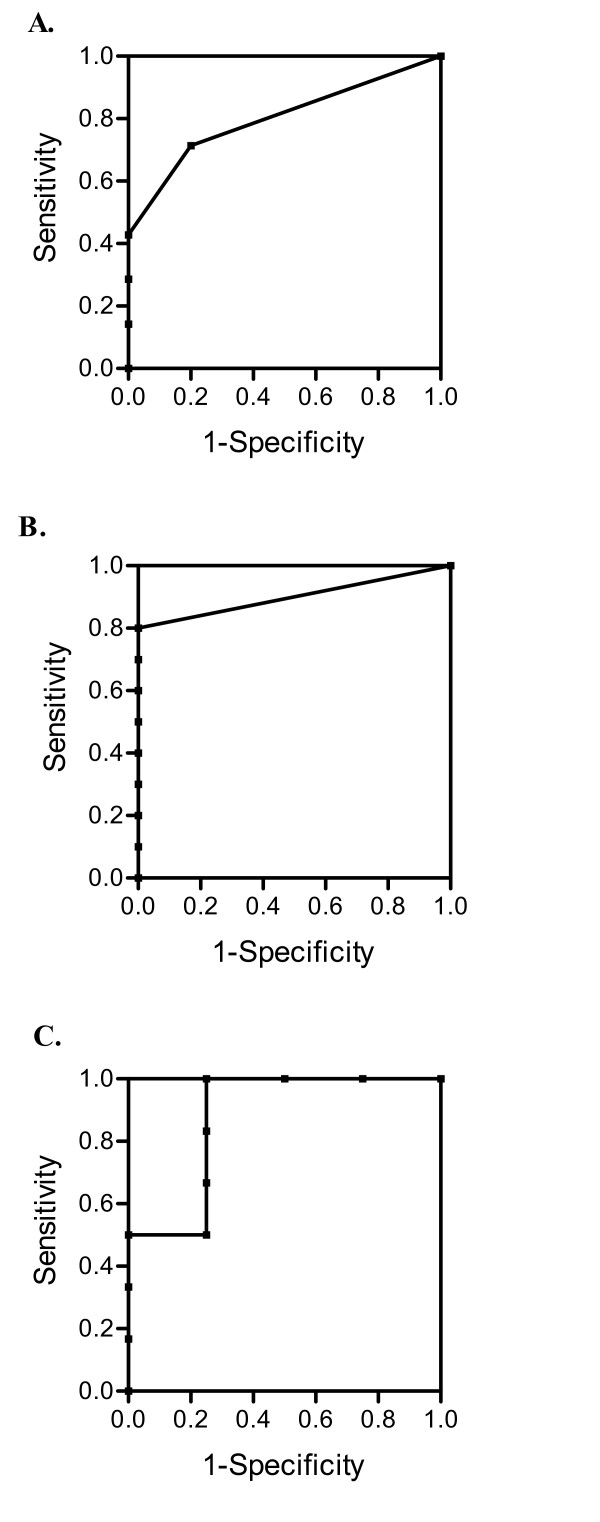
**IFN-gamma secretion as measured by ELISPOT accurately detects robust T cell immunity**. ROC curves were plotted to assess the diagnostic performance of the ELISPOT assay for (A) HER-2/neu ICD specific cellular immunity (n = 18), (B) tt specific cellular immunity (n = 12), and (C) CMV specific cellular immunity (n = 12). Samples with antigen specific circulating antibodies were considered to have positive immunity.

ROC curves were plotted to assess sensitivity and specificity of the ELISPOT assay, using presence of antigen specific antibodies as a marker of successful immunization. Results show that ELISPOT did not significantly detect low level immunity to HER-2/neu or tt. Precursor frequency is not a significant (p = 0.09) indicator of immunity to the HER-2/neu ICD protein with an AUC of 0.800 (Fig. 4A) and sensitivity of 64%. A subset (n = 12) of these patients was assessed by ELISPOT assay for cellular immunity to antigens tt and CMV. Results show that precursor frequency is not a significant indicator of moderate level cellular immunity to tt (AUC = 0.900, p = 0.09) (Fig. 4B), but that ELISPOT will significantly (p = 0.016) predict robust T cell immunity to CMV with an AUC of 0.844 (Fig. 4C). The sensitivity for the ELISPOT tt and CMV assays were 67% and 88%, respectively. Thus, the ELISPOT assay is most accurate when assessing robust T cell immunity.

There were few false positives in this population. That is, patients without an antibody response usually lacked a T cell response as well, by either T cell assay method. A cutoff of 2.0 for SI for the proliferation assay for HER-2/neu specific CD4+ T cells accurately identified 78% of seropositive and seronegative samples, with 2 false positives and 4 false negatives. The HER-2/neu ELISPOT accurately identified 56% of seropositive and seronegative samples, with 3 false positives and 5 false negatives. The tt proliferation assay correctly identified all samples, and the tt ELISPOT 67%, with 4 false negatives. The CMV proliferation assay correctly identified 88% with 2 false positive samples, and the CMV ELISPOT correctly identified 92% of samples, with 1 false negative.

## Discussion

While infectious disease antigens, in general, stimulate a very robust T cell immune response that can easily be detected using a variety of assays, immunity to tumor antigens is generally low level. For this reason, to evaluate the immune response against cancer antigens, assays are needed that can detect a broad range of T cell responses i.e. from innate to induced immunity. We focused on evaluating the sensitivity of two commonly used methods for assessing tumor specific immunity; the proliferation of T cells after antigen exposure measured by the incorporation of tritiated thymidine and IFN-gamma ELISPOT. Compared to other methods of T cell assessment, such as flow cytometry or tetramer analysis, these assays are technically simple, easy to perform for all levels of lab personnel, require smaller numbers of cells, and reagents which are readily available and inexpensive.

Very few studies have attempted to determine the sensitivity and specificity of assays that measure cellular immune responses after vaccination. A major problem preventing such an analysis is the definition of a parameter that predicts that immunity has been successfully elicited. We used the development of humoral immunity to indicate that tumor antigen and foreign antigen vaccinations were effective in stimulating a detectable immune response. Humoral immunity has long been used as an accepted measure of successful immunization. Presence of antigen specific antibodies indicates exposure to antigen and as such is considered proof of immunity (via natural exposure or vaccination) to common infectious diseases such as measles and influenza [6,7]. Vaccine development for infectious disease continues to rely on antibody presence as an indicator not only of exposure to antigen [8], but as a surrogate of protection against disease. Indeed, antigen specific humoral immunity is an indicator for the development of an antigen specific T cell response. In one study, T cell responses measured by IFN-gamma ELISPOT were significantly associated with antibody responses induced by vaccination against *P. falciparum *protein [9]. A recent population-based analysis of immune responses to MMR vaccination found that an antigen specific antibody response was significantly associated with antigen specific T cell proliferation, and that lymphoproliferation was in turn associated with production of IFN-gamma as measured by cytokine ELISA [7]. Thus, although not a perfect measure or "gold standard", humoral immunity can be used as a meaningful determinate of effective immunization by which the T cell assays could be assessed and compared.

Antibody responses to tumor associated antigen demonstrate exposure to tumor, and may be significantly associated with antigen specific cellular responses. However, the relationship between presence of antigen specific antibodies, presence of antigen specific T cells, and clinical response in cancer is not as well characterized as the relationship between antibody and T cell responses, and clinical response in infectious diseases such as malaria. Recently, the presence of tumor-infiltrating T cells has recently been associated with clinical response in various cancers, and antigen specific T cells have been shown necessary for tumor destruction [10-12]. Thus, direct assay of T cell responses, both in terms of measuring quantities and frequencies of T cells and in terms of characterizing T cell function, remains an essential component in elucidating the relationship between immune responses and clinical outcome, and in the immunologic monitoring of clinical trials.

Due to the large number of cells required to perform the proliferation and ELISPOT assays, not all of the samples contained sufficient quantity to perform both assays. It is interesting to note that although the ELISPOT assay for response to CMV tested only 12 samples, compared to the 18 samples tested for CMV responses by proliferation assay, both methods produced results which predicted positive humoral immunity to CMV. In contrast, all 27 samples were tested for HER-2/neu specific responses using both ELISPOT and proliferation assays, and only the proliferation assay produced results which were a significant predictor of antibody immunity. It is possible that the lack of concordance between tt specific results by ELISPOT and tt specific antibody immunity is due to the difference in samples size between T cell assays, as only 12 samples were of sufficient quantity to obtain results by ELISPOT, compared to the 18 available for testing by proliferation assay. Similarly, cell quantities limited the ELISPOT assay to measurement of only IFN-gamma. This may in part explain the ELISPOT's lack of sensitivity in determining low level responses. Although increasing the breadth of cytokine analysis to include measurement of other cytokine responses such as IL-4 or IL-5 may lead to greater sensitivity, the measurement of IFN-gamma alone is standard for most clinical trials.

## Conclusion

The antigens we chose to evaluate, HER-2/neu, tt, and CMV, represented a broad range of responses, from low level to robust. As the majority of patients developed humoral immunity to both HER-2/neu and tt we were able to evaluate the two cell based analytic methods for the ability to detect low level T cell immunity. Antigen specific T cell proliferation discriminated a broader range of responses than ELISPOT. There have been few studies directly comparing T cell assays. One recent study, Karlsson et al in 2003, compared ELISPOT to cytokine flow cytometry and found a lack of association between results when considering low level responses, and that the ELISPOT assay performed better in detecting such responses [13]. Other groups compared thymidine incorporation proliferation assays to BrdU ELISA [14] or CFSE proliferation assay [15]. The first study found the BrdU ELISA performance superior to traditional proliferation assay in detecting low level responses; however, the study involved only cells obtained from rat lymph nodes, not human PBMC. The second study specifically looked at rare antigen specific T cells in human PBMC and found the CFSE assay had superior sensitivity within a small sample set. The only study available which evaluates ELISPOT and proliferation assays found that the proliferation assay was not sensitive enough to detect CA-125 specific T cells in 4 healthy controls or 3 patients with ovarian cancer [16]. Data presented here, on a much larger number of individuals who have documented evidence of successful immunization, would suggest that there are marked differences in the ability of two of the most common cellular immunologic monitoring methods in measuring different levels of T cell immunity. These considerations should be taken into account when choosing analytic methods for assessing the development of T cell immunity, particularly when evaluating tumor specific immune responses which are, for the most part, of low to moderate magnitude.

## Methods

### Subjects

Twenty-seven women with Stage II, III, or IV HER-2/neu overexpressing breast cancer with no evidence of disease after standard therapy, or stage IIC HER-2/neu overexpressing ovarian cancer in complete remission, were enrolled on a phase I study of a HER-2/neu intracellular domain (ICD) protein based vaccine [5]. Median subject age was 49 years (35–76). The study was approved by both the United States Food and Drug Administration and the University of Washington Institutional Review Board. Patients received intradermal (id.) vaccinations with the ICD protein monthly for 6 months. All patients received a tetanus toxoid (tt) booster vaccination prior to beginning immunization with the HER-2/neu protein vaccine. Blood was obtained for assessment of immunologic responses after the last vaccination. After processing, PBMC samples were frozen and stored until time of assay. Samples from all 27 patients were available for analysis of HER-2/neu specific T cell response by a proliferation assay that has been previously described [14]. As immunologic assays were prioritized based on number of available cells, samples from 18 of the 27 were available for analysis of tt and CMV specific responses by proliferation assay and for analysis of HER-2/neu specific response by ELISPOT. Samples from 12 of the 27 were available for analysis of tetanus toxoid and CMV specific response by ELISPOT. All 27 of the samples were run through the proliferation assay for HER-2/neu T cell responses.

### Modified limiting dilution tritiated thymidine incorporation (proliferation) assay

The CD4+ proliferation assay was modified from a previously described [17] assay for CD8+ T cells. The assay is performed in 24-well replicates. PBMC were isolated from heparinized peripheral blood by Ficoll/Hypaque-density gradient centrifugation. Briefly, 2 × 10^5 ^PBMCs/well were plated into 96-well plates in 24-well replicates in media consisting of equal parts EHAA 120 (Biofluids, Rockville, MD) and RPMI 1640 (Life Technologies, Inc., Gaithersburg, MD) with L-glutamine, penicillin/streptomycin, 2 ME, and 10% AB serum (ICN Flow, Costa Mesa, CA) in the presence or absence of 25 ug/ml HER-2/neu ICD protein (Corixa Corp., Seattle, WA), 0.5 U/ml tetanus toxoid (Lederle, Pearl river, NY), or 2.5 ug/ml CMV lysate (EastCoast Bio, North Berwick, ME). After 5 days, wells were pulsed with 1 uCi of [^3^H]thymidine for 8–10 h and counted. Stimulation index (SI) is defined as the mean CPM of the response of the antigen-stimulated cells divided by the mean of the response of cells cultured without antigen. Positive and negative controls were run on each plate. Phytohemagglutinin (Sigma, St. Louis, MO) incubated with patient T cells was used as a positive control to assess the ability of the T cells to respond to mitogen. Media alone was used as a negative control in addition to no antigens wells, and myoglobin (Chemicon, Temecula, CA) was used as an irrelevant protein. A reference population of 30 age-matched volunteer donors was previously used to establish base line response to all antigens [5]. The mean and 3 SDs for the normal population resulted in an SI of 1.98, therefore an SI greater than 2.0 was considered consistent with an immunized response.

### IFN-gamma enzyme-linked immunosorbent spot (ELISPOT) assay

An IFN-gamma ELISPOT assay was used to determine precursor frequencies of antigen-specific T lymphocytes as previously described [18]. Briefly, on day 1, 2.5 × 10^5 ^PBMCs/well were plated into 96-well plates in six-well replicates in 200 ul of RPMI-1640 containing L-glutamine, penicillin, streptomycin, and 10% AB serum (T-cell medium) in the presence or absence of 10 μg/ml peptide antigen (Corixa), CMV lysate (EastCoast Bio) or 0.5 U/ml tetanus toxoid (Lederle). The cells were incubated at 37°C at 5% CO_2_. On day 5, IL-2 was added to 10 U/ml. On day 8, 2.5 × 10^5^/well irradiated autologous PBMCs and 10 ug/ml antigens were added. Also on day 8, nitrocellulose-backed 96-well plates (NC-plates) were coated with 10 ug/ml anti-IFN-gamma Ab in PBS at 50 ul/well. On day 9 the NC-plate was washed three times with PBS and blocked for 2 hours with PBS containing 2% BSA, followed by three washes with PBS. On day 9, the cells were gently resuspended, pooled, centrifuged, and the media was replaced. The cells were transferred into the NC-plate in a volume of 100 ul/well in T-cell medium. The NC-plate was incubated at 37°C for a further 20–24 hours followed by washing three times using PBS containing 0.05% Tween-20. The plate was then incubated for 2.5 hours at room temperature in 50 ul/well PBS containing 5 ug/ml biotinylated anti-IFN-gamma Ab, washed three times with PBS, and further incubated with 100 ul/well streptavidin-alkaline phosphatase at a dilution of 1:1,000 in PBS for 2 hours at room temperature. After washing three times in PBS, the plate was incubated with 100 ul/well AP-colorimetric substrate for 20–30 minutes, rinsed with cool tap water, and allowed to dry completely. After assay completion, resultant spots were enumerated using a dissecting microscope. Precursor frequencies were calculated by subtracting the mean number of spots obtained from the no-antigen control wells from the mean number obtained in the experimental wells. A positive response was defined as a precursor frequency that was both significantly (p < 0.05) greater than the mean of control no-antigen wells and detectable (i.e., >1:100,000). In addition to non-antigen wells, myoglobin (Chemicon) and media alone wells were included as an irrelevant protein and negative control. Phytohemagglutinin (Sigma) incubated with patient T cells at a concentration of 5 ug/ml was used as a positive control for the ability of T cells to respond to antigen.

### ELISA for detection of antigen specific antibodies

Humoral immunity to HER-2/neu, tt, and CMV was assessed using a standardized and validated enzyme-linked immunosorbant assays (ELISA) that have been previously described [19]. Briefly, 96-well microtiter plates (Dynex Technologies, Inc, Chantilly, VA) were coated with antigen in alternating columns. Serially diluted, purified human IgG (Sigma) provided a standard curve. Plates were incubated overnight at 4°C. All wells were then blocked with 100 uL/well of a filtered buffer of 10% phosphate-buffered saline (PBS)/1% bovine serum albumin (BSA; Sigma), 100 uL/well, and incubated at room temperature on a rocker for 4 hours. Plates were washed four times with 10% PBS/0.5% Tween (Amersham Biosciences, Piscataway, NJ) before addition of patient sera. After serum incubation, plates were washed four times with PBS/Tween and goat antihuman IgG-horseradish peroxidase (HRP) conjugate (Zymed Laboratories, South San Francisco, CA) added at a dilution of 1:50,000 (50 uL/well) and incubated for 45 minutes at room temperature on rocker. After a final PBS/Tween wash, developing reagent was added (75 uL/well) and color reaction assessed at an optical density (OD) of 640 nm until the well containing the standard at a concentration of 0.16 ug/mL evaluated at 0.3 OD. Reaction was then stopped with 75 uL/well 1N HCL and read at OD of 450 nm. The OD of each serum dilution was calculated as the OD of the antigen-coated wells minus the OD of the PBS/BSA-coated wells. Values for each delta OD were calculated from the log-log equation of the line for the standard curve on each plate, as plotted by SOFTmax version 2.3 for Macintosh (Molecular Devices Corp, Sunnyvale, CA). Positive and negative controls were run on every plate. A sample from a patient with HER-2/neu overexpressing breast cancer was used as a positive control. Media alone and a sample from a healthy donor were used as negative controls. A sample was defined as positive if the value was greater than the mean and two standard deviations of the previously analyzed reference population, 1.13 ug/ml for the HER-2/neu assay, 9.98 ug/ml for the tt assay and 200 ug/ml for the CMV assay.

### Statistical analysis

Differences in magnitude of T cell responses to different antigens were measured by one-way ANOVA. The relationship between antibody and T cell response was analyzed by linear regression. Analytic performance of the proliferation assay and ELISPOT assay was evaluated by plotting receiver operating characteristic (ROC) curves of results and estimating area under the curve (AUC). All analyses were performed using SPSS 13.0 for PC (SPSS, Chicago, IL).

## Abbreviations

AUC- Area under the curve.

CMV- Cytomegalovirus.

CV- Coefficient of variation.

ICD- Intracellular domain.

mLDA- Modified limiting dilution assay.

ELISPOT- Enzyme-linked immunosorbent spot.

ROC- Receiver operating characteristic.

SI- Stimulation index.

tt- Tetanus toxoid.

## Authors' contributions

VG performed antibody assays and statistical analysis. CdR, MS and BM performed all T cell assays. MLD conceived of the study, and participated in its design and coordination. All authors helped to draft the manuscript and read and approved the final manuscript.

## References

[B1] Knutson KL, dela Rosa C, Disis ML (2006). Laboratory analysis of T-cell immunity. Front Biosci.

[B2] Ochsenbein AF, Pinschewer DD, Sierro S, Horvath E, Hengartner H, Zinkernagel RM (2000). Protective long-term antibody memory by antigen-driven and T help-dependent differentiation of long-lived memory B cells to short-lived plasma cells independent of secondary lymphoid organs. Proc Natl Acad Sci USA.

[B3] Nielsen CH, Hegedus L, Leslie RG (2004). Autoantibodies in autoimmune thyroid disease promote immune complex formation with self antigens and increase B cell and CD4+ T cell proliferation in response to self antigens. Eur J Immunol.

[B4] Gnjatic S, Atanackovic D, Jager E, Matsuo M, Selvakumar A, Altorki NK, Maki RG, Dupont B, Ritter G, Chen YT, Knuth A, Old LJ (2003). Survey of naturally occurring CD4+ T cell responses against NY-ESO-1 in cancer patients: correlation with antibody responses. Proc Natl Acad Sci USA.

[B5] Disis ML, Schiffman K, Guthrie K, Salazar LG, Knutson KL, Goodell V, dela Rosa C, Cheever MA (2004). Effect of dose on immune response in patients vaccinated with an HER-2/neu intracellular domain protein-based vaccine. J Clin Oncol.

[B6] Cheong HJ, Song JY, Park JW, Yeon JE, Byun KS, Lee CH, Cho HI, Kim TG, Kim WJ (2006). Humoral and cellular immune responses to influenza vaccine in patients with advanced cirrhosis. Vaccine.

[B7] Dhiman N, Ovsyannikova IG, Oberg AL, Grill DE, Jacobson RM, Poland GA (2005). Immune activation at effector and gene expression levels after measles vaccination in healthy individuals: a pilot study. Hum Immunol.

[B8] Stoute JA, Heppner DG Jr, Mason CJ, Siangla J, Opollo MO, Kester KE, Vigneron L, Voss G, Walter MJ, Tornieporth N, Cohen JD, Ballou WR (2006). Phase 1 safety and immunogenicity trial of malaria vaccine RTS,S/AS02A in adults in a hyperendemic region of western Kenya. Am J Trop Med Hyg.

[B9] Sun W, Edelman R, Kanesa-Thasan N, Eckels KH, Putnak JR, King AD, Houng HS, Tang D, Scherer JM, Hoke CH, Innis BL (2003). Vaccination of human volunteers with monovalent and tetravalent live-attenuated dengue vaccine candidates. Am J Trop Med Hyg.

[B10] Zhang L, Conejo-Garcia JR, Katsaros D, Gimotty PA, Massobrio M, Regnani G, Makrigiannakis A, Gray H, Schlienger K, Liebman MN, Rubin SC, Coukos G (2003). Intratumoral T cells, recurrence, and survival in epithelial ovarian cancer. N Engl J Med.

[B11] Ahmed N, Ratnayake M, Savoldo B, Perlaky L, Dotti G, Wels WS, Bhattacharjee MB, Gilbertson RJ, Shine HD, Weiss HL, Rooney CM, Heslop HE, Gottschalk S (2007). Regression of experimental medulloblastoma following transfer of HER2-specific T cells. Cancer Res.

[B12] Dudley ME, Wunderlich JR, Robbins PF, Yang JC, Hwu P, Schwartzentruber DJ, Topalian SL, Sherry R, Restifo NP, Hubicki AM, Robinson MR, Raffeld M, Duray P, Seipp CA, Rogers-Freezer L, Morton KE, Mavroukakis SA, White DE, Rosenberg SA (2002). Cancer regression and autoimmunity in patients after clonal repopulation with antitumor lymphocytes. Science.

[B13] Karlsson AC, Martin JN, Younger SR, Bredt BM, Epling L, Ronquillo R, Varma A, Deeks SG, McCune JM, Nixon DF, Sinclair E (2003). Comparison of the ELISPOT and cytokine flow cytometry assays for the enumeration of antigen-specific T cells. J Immunol Methods.

[B14] Maghni K, Nicolescu OM, Martin JG (1999). Suitability of cell colorimetric assays for assessment of CD4+ T cell proliferation: comparison to 5-bromo-2-deoxyuridine (BrdU) ELISA. J Immunol Methods.

[B15] Mannering SI, Morris JS, Jensen KP, Purcell AW, Honeyman MC, van Endert PM, Harrison LC (2003). A sensitive method for detecting proliferation of rare autoantigen-specific human T cells. J Immunol Methods.

[B16] Schultes BC, Whiteside TL (2003). Monitoring immune responses to CA 125 with an IFN-γ ELISPOT assay. J Immunol Methods.

[B17] Disis ML, Grabstein KH, Sleath PR, Cheever MA (1999). Generation of immunity to the HER-2/neu oncogenic protein in patients with breast and ovarian cancer using a peptide-based vaccine. Clin Cancer Res.

[B18] Knutson KL, Schiffman K, Disis ML (2001). Immunization with a HER-2/neu helper peptide vaccine generates HER-2/neu CD8 T-cell immunity in cancer patients. J Clin Invest.

[B19] Goodell V, Disis ML (2005). Human tumor cell lysates as a protein source for the detection of cancer antigen-specific humoral immunity. J Immunol Methods.

